# Case Report: Functional Investigation of an Undescribed Missense Variant Affecting Splicing in a Patient With Dravet Syndrome

**DOI:** 10.3389/fneur.2021.761892

**Published:** 2021-12-06

**Authors:** Peter Sparber, Svetlana Mikhaylova, Varvara Galkina, Yulia Itkis, Mikhail Skoblov

**Affiliations:** ^1^Laboratory of Functional Genomics, Research Centre for Medical Genetics, Moscow, Russia; ^2^Medical Genetics Department, Russian Children's Clinical Hospital, Moscow, Russia; ^3^Clinical Department, Research Centre for Medical Genetics, Moscow, Russia; ^4^Laboratory of Inherited Metabolic Disorders, Research Centre for Medical Genetics, Moscow, Russia

**Keywords:** epilepsy, Dravet syndrome, medical genetics, functional analysis, splicing, molecular pathogenesis

## Abstract

Pathogenic variants in the *SCN1A* gene are associated with a spectrum of epileptic disorders ranging in severity from familial febrile seizures to Dravet syndrome. Large proportions of reported pathogenic variants in *SCN1A* are annotated as missense variants and are often classified as variants of uncertain significance when no functional data are available. Although loss-of-function variants are associated with a more severe phenotype in *SCN1A*, the molecular mechanism of single nucleotide variants is often not clear, and genotype-phenotype correlations in *SCN1A*-related epilepsy remain uncertain. Coding variants can affect splicing by creating novel cryptic splicing sites in exons or by disrupting exonic cis-regulation elements crucial for proper pre-mRNA splicing. Here, we report a novel case of Dravet syndrome caused by an undescribed missense variant, c.4852G>A (p.(Gly1618Ser)). By midigene splicing assay, we demonstrated that the identified variant is in fact splice-affecting. To our knowledge, this is the first report on the functional investigation of a missense variant affecting splicing in Dravet syndrome.

## Introduction

Dravet syndrome (DS), also known as severe myoclonic epilepsy (SMEI) in infancy, is a developmental and epileptic encephalopathy (DEE) characterized by intractable childhood-onset seizures, neurodevelopmental delay, and other neurological impairments (1). With overall incidence ranging from 1:15,700 in the United States and 1:40,000 according to the Orphanet database (2), DS is considered to be one of the most common causes of genetic epilepsy in infants (3). The main cause of DS is heterozygous loss-of-function (LoF) pathogenic variants in the *SCN1A* gene that encodes the alpha subunit of type I voltage-gated sodium channel (Nav1.1) (4). In a small fraction of patients with DS phenotype, pathogenic variants in other genes, including *PCDH19, GABRA1*, and *STXBP1*, were shown to be causative (5). *SCN1A* expression is restricted to GABA-ergic inhibitory interneurons that control neuronal excitability, and loss of Nav1.1 is assumed to lead to uncontrolled firing and epileptic activity (6–8).

Given the fact that *SCN1A* haploinsufficiency is the main molecular mechanism leading to DS, protein-truncation variants (nonsense, frameshift, and splice-site variants) are, in general, associated with a more severe epileptic phenotype (9). However, there are hundreds of missense variants that are associated with DS in the literature lacking any functional data (10). The absence of functional data for the majority of missense variants makes it challenging for performing valid genotype-phenotype studies in *SCN1A*-related epileptic disorders.

Here, we report a novel case of a 2-year-old patient who was clinically diagnosed with DS. Genetic analysis revealed an undescribed missense variant in the *SCN1A* gene, c.4852G>A (p.(Gly1618Ser)). We showed by midigene splicing assay that the variant is in fact splice-affecting, leading to two independent splicing alterations and LoF effects, thus confirming the diagnosis.

## Case Description

**Figure 1:**
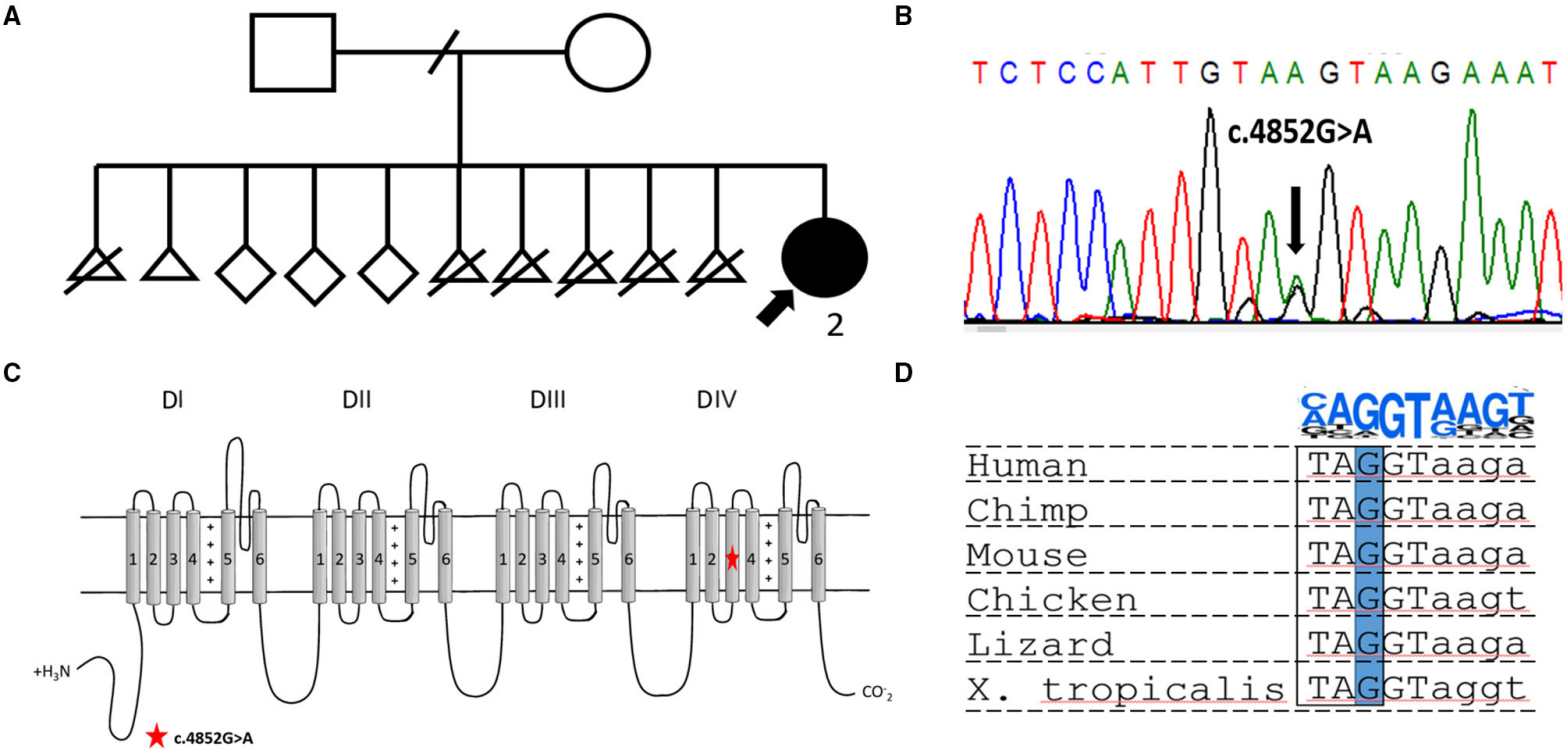
**(A)** Pedigree of the reported family. The proband is indicated by a bold arrow. **(B)** Electropherogram showing the heterozygous variant NM_001165963.2:c.4852G>A [p.(Gly1618Ser)] confirmed in the proband by Sanger sequencing. **(C)** Scheme of Nav1.1 channel structure. The location of the novel missense variant is indicated by a red star. **(D)** Multiply alignment showing the conservation of the affected nucleotide in different vertebrates. On the top is a position weight matrix of the site of the splicing donor.

The proband, a 2-year-old Russian girl at the time of the last clinical evaluation, was admittedto the neurological department with repeated, prolonged myoclonic, and generalized seizures responsive only to intravenous injection of diazepam. She was the fourth child of healthy parents from a non-consanguineous marriage. The proband has three healthy siblings (Figure 1A). During the pregnancy, a risk of miscarriage was observed at 12 weeks of gestation. Delivery and neonatal period were unremarkable. At the age of 4 months, during obstructive bronchitis, she developed an absence seizure with apnea 3–5 s long that repeated daily afterward. At the age of 5 months, during hot water bathing, the proband had a prolonged generalized myoclonic seizure for 40 min that was responsive only to diazepam injection. Similar episodes repeated every 7–10 days without any provoking factors.

On brain magnetic resonance imaging (MRI), periventricular leukomalacia was noted and was considered as a result of ischemic brain injury with no relevance to the epileptic phenotype of the patient. Routine electroencephalography (EEG) did not show any epileptiform activity in the interictal period. Antiepileptic therapy included carbamazepine (300 mg/day), topiramate (87.5 mg/day), and clonazepam (0.75 mg/day), which had no effect on seizure frequency and duration. In the evaluation, the proband had prolonged myoclonic, tonic-clonic, and atonic seizures once a week. Early motor milestones were normal, but language development was delayed. Neurological examination at the age of 2 revealed moderate hypotonia with brisk tendon reflexes and mild gait ataxia. Based on the clinical picture, the proband was diagnosed with DS.

## Genetic and Bioinformatic Analysis

Targeted sequencing of 587 genes associated with neurodevelopmental disorders, such as the *SCN1A* gene, along with other DS associated genes, *PCDH19* and *STXBP1*, was performed. DNA analysis revealed a previously undescribed missense variant in the *SCN1A* gene, NM_001165963.2:c.4852G>A (p.(Gly1618Ser)). Sanger sequencing confirmed the variant in the proband (Figure 1B), but segregation analysis was not performed because of the fact that the father was unavailable for biological sample collection and the mother declined genetic testing for herself and other healthy siblings. The variant is absent in the gnomAD database and predicted to be deleterious by several bioinformatic predictors, such as PolyPhen2, SIFT, and BadMut (11–13). According to the ACMG/AMP guidelines (14), it was classified as a variant of uncertain significance (VUS).

The undescribed missense variant is predicted to change a conservative amino acid residue located in the S3 transmembrane segment in domain IV of Nav1.1 (Figure 1B). Previous studies have shown that missense substitutions located in the pore-forming part are frequently associated with complete LoF and, therefore, lead to a severe epileptic phenotype (15). In other cases, for missense variants, it is nearly impossible to predict the phenotypic outcome based only on genetic data. At the DNA level, the c.4852G>A variant changes the last nucleotide in exon 28 of the *SCN1A* gene (Figures 1C,D). Therefore, we assumed that the variant could affect splicing. Bioinformatic analysis using SpliceAI (16) predicted donor loss (Δ score.53) and activation of a cryptic exonic donor site at position −4 (Δ score.83).

## Functional Analysis

**Figure 2:**
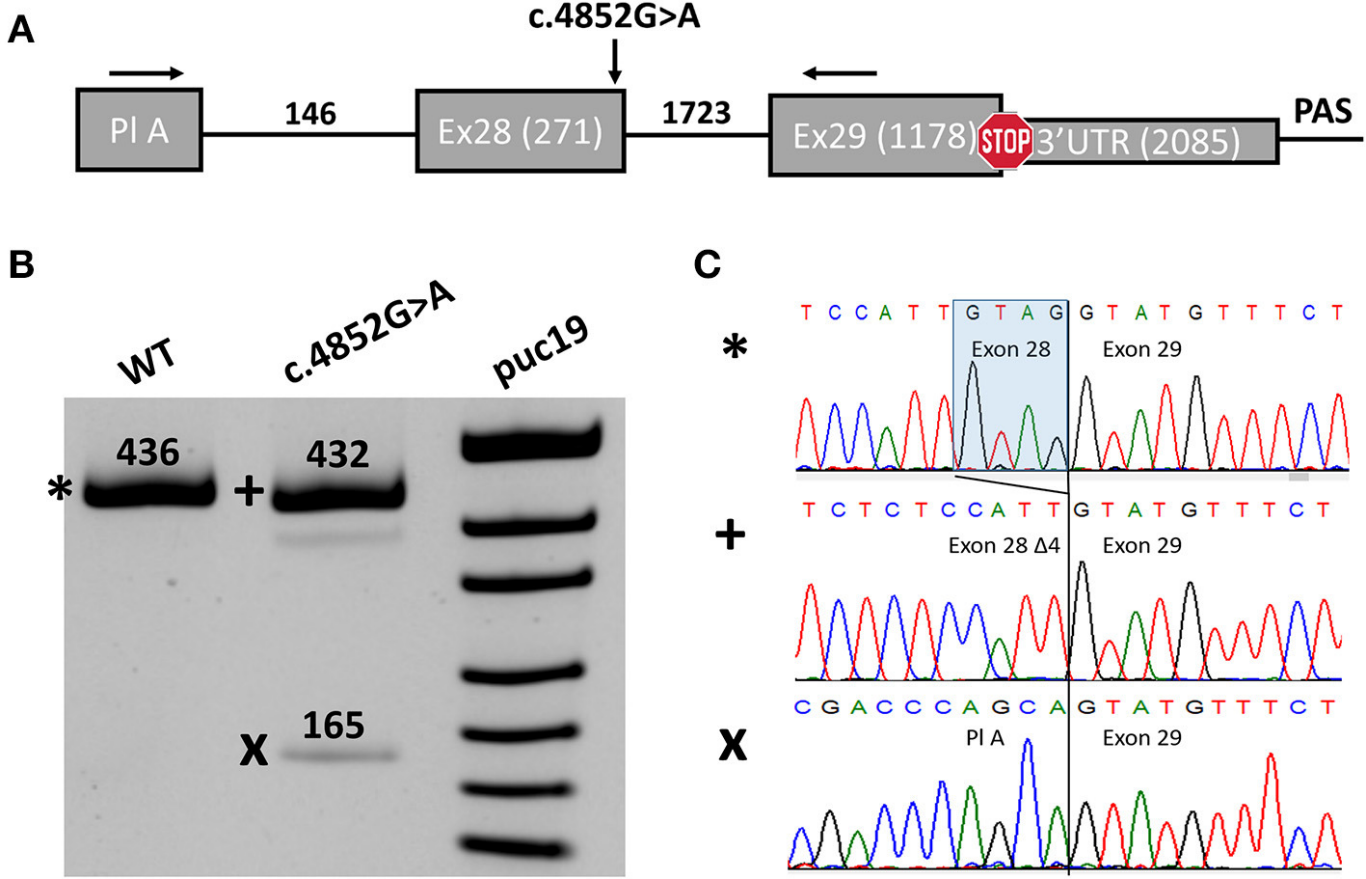
Midigene splicing assay results. **(A)** Scheme of the midigene plasmid used for splicing analysis with numbers indicating the length of exons and introns. The location of primers used for RT-PCR and the investigated variant are shown with black arrows. Pl A, plasmid exon A. **(B)** PAGE urea electrophoresis of RT-PCR products. *, Wild type isoform; +, isoform with four-nucleotide deletions of exon 28; x, isoform with exon 28 skipping. WT, wild type. **(C)** Sanger sequencing of the corresponding isoforms.

In order to investigate the potential impact of the c.4852G>A variant on splicing, we performed a midigene splicing assay. The genomic region containing exon 28 with 146 nucleotides of intron 27, intron 28, and exon 29 of the *SCN1A* gene was amplified from the genomic DNA of the proband and cloned into a pSpl3-Flu splicing vector (Figure 2A) (17). Wild-type (WT) and mutant (MUT) plasmids were transfected separately to HEK293T, and 48 h post transfection RT-PCR was performed. The WT plasmids demonstrated a normal splicing pattern with the inclusion of both exons 28 and 29 (Figure 2B).

We detected two abnormal isoforms when analyzing the splicing pattern of the MUT plasmid (Figure 2B). In the major isoform, we observed a four-nucleotide deletion due to the activation of a cryptic donor splicing site (Figure 2C). The deletion caused frameshift and formation of a premature termination codon (PTC) (p.(Gly1618Cysfs^*^6)). Sanger sequencing of the second isoform revealed skipping of exon 28 (Figure 2C), which also leads to the frameshift in CDS (p.(Asn1528Valfs^*^7)). The splicing alteration affected the penultimate exon of the *SCN1A* gene. In both cases, the PTC is formed in the last exon and, therefore, is not predicted to induce non-sense-mRNA decay (NMD) (18, 19). Nevertheless, the predicted truncated protein, even if translated, could not function properly as it lacks the majority of the IV domain of Nav1.1 including pore-forming residues. Therefore, we conclude that the c.4852G>A variant leads to complete LoF and could be reclassified as likely pathogenic (PM2, PS3).

## Discussion

Pathogenic variants in the *SCN1A* gene are associated with a spectrum of epilepsy phenotypes ranging in severity from familial febrile seizures (FSs) on the mild end to DS on the other. According to the *SCN1A* mutation database, almost 50% of reported pathogenic variants in this gene are missense substitutions (10). LoF variants (non-sense, frameshift, and splice-site variants) are frequently found in patients with more severe phenotypes and are evenly distributed in different exons, as they are predicted to activate NMD and, therefore, lead to loss of expression from one allele. On the other hand, missense variants are more often found in patients with milder phenotypes, such as mild generalized epilepsy (GE) and/or FS. In contrast, with LoF variants, the frequency of missense variants decreases with an increase in severity. Analysis of the distribution of missense variants across different Nav1.1 domains shows that variants linked to DS are enriched in the pore-forming region (S5–S6) (10). Such substitutions completely disturb the kinetics of the Nav1.1 channel, as they prevent sodium current from entering the cell (20).

The introduction of next-generation sequencing (NGS) to clinical practice has revolutionized the field of genetic-based epilepsy and has improved the diagnostic yield dramatically (21, 22). However, with more genetic tests performed, the interpretation has become more and more challenging for newly described variants. With more than 2,000 pathogenic variants in *SCN1A* reported in the professional version of the HGMD database, for the majority of missense variants, there is no functional characterization. The gold standard for functional tests in channelopathies is the patch clamp technique where ionic currents are measured in the context of the variant of interest (20, 23). Although very informative for the investigation of channel function, patch clamp remains low-scaled, time-consuming, and is not routinely performed.

Several studies have demonstrated that a large fraction of all disease-causing variants may influence normal pre-mRNA splicing (24, 25). A recent high-throughput study revealed that ~10% out of 5,000 pathogenic missense variants reported in the literature leads to exon skipping (26). Taking into account that only exon skipping was investigated and that other splicing alterations, such as cryptic splice-site activation and intron retention, could not be assessed, the true rate of missense variants affecting splicing could even be higher, which means that many missense variants reported in the literature could, in fact, affect splicing and are misannotated.

Ribonucleic acid (RNA) analysis using patient-derived samples is the most informative approach for the investigation of splicing alterations (27). Unfortunately, in many cases, when analyzing variants in a gene with a tissue-specific expression, such as *SCN1A*, the collection of a relevant biological sample is impossible. In such cases, the minigene splicing assay could be performed as an alternative. Minigene splicing assay is a well-established method that could be routinely used and has demonstrated high concordance with RT-PCR results (28). Functional analysis is of great importance given the fact that several phenotypes are linked to the *SCN1A* gene with different molecular mechanisms of channel dysfunction (29, 30). Moreover, in the absence of segregation data, functional tests are often the only option for the reclassification of an undescribed variant. Proper understanding of the molecular defect is crucial not only for proper genetic counseling but is also necessary for developing tailored therapeutic approaches in genetic epilepsy.

In this report, the diagnosis of DS was made based solely on the severe epileptic phenotype with poor response to antiepileptic medications. When performing a genetic test, a novel missense variant in *SCN1A* was observed. However, the predicted amino acid substitution in the lack of segregation data was not sufficient enough to explain or to confirm the diagnosis of the patient. Bioinformatic analysis predicted a possible effect on splicing that was shown by using midigene splicing assay. The reported patient fully fits the diagnostic criteria of DS, and the epileptic phenotype is similar to that of patients with other reported LoF variants (2). Thereby, we exclude the inconsistency between the genotype and phenotype data and help the clinician in performing straightforward genetic counseling and proper risk prognosis in the family.

Overall, we report a newly described missense variant in a patient with a clinical diagnosis of DS. Midigene splicing assay confirmed that the c.4852G>A variant affects splicing and leads to complete LoF of Nav1.1. This study expands the mutational spectrum of *SCN1A* related DS and delineated the patient phenotype on the molecular level. This report highlights the importance of functional validation in clinical practice and its role in robust genotype-phenotype correlations. To our knowledge, this is the first report on a splice-affecting missense variant in DS.

## Materials and Methods

### Subjects

The proband underwent a detailed clinical examination at the Russian Children's Clinical Hospital in Moscow. Genetic analysis was performed at the Research Center for Medical Genetics, Russia.

All research participants gave informed consent (or responsible consent form for infant proband) for the clinical examination and publication of their anonymized data. The study was performed in accordance with the Declaration of Helsinki and approved by the Institutional Review Board of the Research Center for Medical Genetics., Russia.

### Genetic Analysis

DNA was extracted from peripheral blood mononuclear cells using the standard phenol-chloroform method. Genetic analysis was performed at the Research Center for Medical Genetics by the next-generation sequencing of a gene panel containing 587 genes. The sequencing library was prepared with The Ion AmpliSeq™ Library Kit 2.0 and sequenced on an Ion Torrent S5 system with a minimum coverage of 140X. Reads were aligned to the human reference genome (Hg19) with BWA and filtered based on frequency and annotation. The candidate variant in the *SCN1A* gene identified in the proband was confirmed by Sanger sequencing. For the *SCN1A* gene, RefSeq accession numbers NG_011906.1 and NM_001165963.4 were used.

### Midigene Splicing Assay

The last two exons of the *SCN1A* (28 and 29) gene along with intron 28 and the adjust 146 nucleotides of intron 27 were amplified from genomic DNA using a high-fidelity DNA polymerase. The PCR product was cloned to a pSpl3-Flu splicing vector as previously described (31). Three polyadenylation signals were introduced after the end of exon 29 for transcription termination. Due to the large insertion size (5,257 bp), we named the resultant vector a “midigene” as in Sangermano et al. (32). HEK293T cells were plated in a 24-well plate and transfected with the SCN1A midigene at ~80% cell confluency with 0.75 μg per well by using the calcium phosphate method (33). Forty-eight hours post transfection, the cells were harvested, and total RNA was extracted using the TRIzol-based method and reverse transcribed. RT-PCR using primers aligned to the plasmid exon and exon 29 of the *SCN1A* gene was performed, and PCR products were visualized by denaturing urea PAGE with subsequent Sanger sequencing.

## Data Availability Statement

The datasets presented in this article are not readily available due to ethical and privacy restrictions. Requests to access the datasets should be directed to the corresponding author.

## Ethics Statement

The studies involving human participants were reviewed and approved by Institutional Review Board of the Research Center for Medical Genetics. Written informed consent to participate in this study was provided by the participants' legal guardian/next of kin. Written informed consent was obtained from the minor(s)' legal guardian/next of kin for the publication of any potentially identifiable images or data included in this article.

## Author Contributions

PS: conceptualization, methodology, design, writing—original draft preparation. SM, VG, and YI: methodology, data curation. MS: conceptualization, methodology, supervision, writing—review and editing. All authors have read and agreed to the published version of the manuscript.

## Funding

This research was carried out within the state assignment of the Ministry of Science and Higher Education of the Russian Federation for RCMG. The reported study was funded by RFBR. Project number: 20-315-90042.

## Conflict of Interest

The authors declare that the research was conducted in the absence of any commercial or financial relationships that could be construed as a potential conflict of interest.

## Publisher's Note

All claims expressed in this article are solely those of the authors and do not necessarily represent those of their affiliated organizations, or those of the publisher, the editors and the reviewers. Any product that may be evaluated in this article, or claim that may be made by its manufacturer, is not guaranteed or endorsed by the publisher.
